# Impact of Meteorological Conditions on the Dynamics of the COVID-19 Pandemic in Poland

**DOI:** 10.3390/ijerph18083951

**Published:** 2021-04-09

**Authors:** Bogdan Bochenek, Mateusz Jankowski, Marta Gruszczynska, Grzegorz Nykiel, Maciej Gruszczynski, Adam Jaczewski, Michal Ziemianski, Robert Pyrc, Mariusz Figurski, Jarosław Pinkas

**Affiliations:** 1 Institute of Meteorology and Water Management-National Research Institute, 01-673 Warsaw, Poland; marta.gruszczynska@imgw.pl (M.G.); grzegorz.nykiel@imgw.pl (G.N.); adam.jaczewski@imgw.pl (A.J.); michal.ziemianski@imgw.pl (M.Z.); robert.pyrc@imgw.pl (R.P.); mariusz.figurski@imgw.pl (M.F.); 2 Centre of Postgraduate Medical Education, School of Public Health, 01-826 Warsaw, Poland; jpinkas@cmkp.edu.pl; 3 Faculty of Civil and Environmental Engineering, Gdansk University of Technology, 80-233 Gdansk, Poland; 4 Central Office of Measures, Time and Frequency Laboratory, 00-137 Warsaw, Poland; maciej.gruszczynski@gum.gov.pl

**Keywords:** COVID-19, SARS-CoV-2, coronavirus, temperature, relative humidity, sunshine, transmission, Poland

## Abstract

Coronavirus disease 2019 (COVID-19) is an infectious disease caused by the novel coronavirus. The role of environmental factors in COVID-19 transmission is unclear. This study aimed to analyze the correlation between meteorological conditions (temperature, relative humidity, sunshine duration, wind speed) and dynamics of the COVID-19 pandemic in Poland. Data on a daily number of laboratory-confirmed COVID-19 cases and the number of COVID-19-related deaths were gatheredfrom the official governmental website. Meteorological observations from 55 synoptic stations in Poland were used. Moreover, reports on the movement of people across different categories of places were collected. A cross-correlation function, principal component analysis and random forest were applied. Maximum temperature, sunshine duration, relative humidity and variability of mean daily temperature affected the dynamics of the COVID-19 pandemic. An increase intemperature and sunshine hours decreased the number of confirmed COVID-19 cases. The occurrence of high humidity caused an increase in the number of COVID-19 cases 14 days later. Decreased sunshine duration and increased air humidity had a negative impact on the number of COVID-19-related deaths. Our study provides information that may be used by policymakers to support the decision-making process in nonpharmaceutical interventions against COVID-19.

## 1. Introduction

Coronavirus disease 2019 (COVID-19) is an infectious disease caused by severe acute respiratory syndrome coronavirus 2 (SARS-CoV-2) [1,2]. The most common COVID-19 symptoms are fever, cough, fatigue, dyspnea and sputum [3]. COVID-19 is a highly contagious disease and one infected person can infect two to three other people [4]. SARS-CoV-2 spreads mainly through human-to-human transmission via respiratory droplets generated when an infected person coughs, sneezes or speaks [1,5]. Fomite transmission by touching a contaminated surface and then touching eyes, nose or mouth also occurs [5]. Moreover, there is a scientific debate on the airborne transmission of SARS-CoV-2 infections (the percentage of infections that occurred through airborne transmission) but scientific evidence is inconclusive [5,6].

On 11 March 2020, the World Health Organization announced the COVID-19 outbreak as a pandemic [7]. However, the dynamics of the COVID-19 pandemic differ across the world. As of 31 March 2021, more than 125 million COVID-19 cases and 2.8 million COVID-19-related deaths have been reported globally [8]. The highest infection and mortality rates have been reported in the US, Brazil and India [8,9]. Moreover, relatively high transmission dynamics of SARS-CoV-2 have been also observed in Europe. Poland is on the list of 15 countries with the highest absolute number of COVID-19 cases in the world [8]. The first laboratory-confirmed COVID-19 case in Poland was reported on 4 March 2020 [10]. As of 31 January 2021, more than 1.5 million COVID-19 cases have been confirmed [8,11].

By the end of 2020, control measures to contain the spread of the COVID-19 pandemic based on social distancing, isolation of confirmed cases, contact tracing and quarantining were introduced [10]. In January 2021, vaccination against COVID-19 began in over 50 countries around the world. Differences in the dynamics of the COVID-19 pandemic across the world result from different mitigation strategies [10,12]. However, more and more studies indicate the role of environmental factors in COVID-19 transmission [13,14,15,16].

Seasonality is a long-recognized attribute of virus infection of humans [17,18,19,20]. Seasonal changes in the transmission of respiratory viruses depend on meteorological factors [13,17,18,19,20]. There is a growing body of evidence on the potential seasonality of COVID-19 [21,22,23]. A modelling study using fine-scaled weather data and global reports of infections predicted that, without intervention, COVID-19 will temporarily decrease during summer, rebound by autumn, and peak next winter [21]. An analysis of 40 sites from 21 countries showed that in temperate sites excluding China 53.1% of annual COVID-19 cases occurred during influenza season [22]. The Susceptible, Exposed, Infectious, Recovered (SEIR) model showed that the cold season in Southern Hemisphere countries caused a 59.7% increase inthe total number of COVID-19 cases, while the warm season in the Northern Hemisphere countries contributed to a 46.4% reduction inthe COVID-19 cases [23].

Some meteorological factors, e.g., temperature or sunlight, may facilitate SARS-CoV-2 destructions and impair the stability of the virus on different types of surfaces [24]. Moreover, weather conditions can influence social behavior, including mobility and the number of people-to-people contacts. Some authors suggest that high temperature, as well as high humidity, mitigates the transmission of COVID-19 [14,15,16]. Data on seasonality of SARS-CoV-2 infections are also limited. The number of studies on weather and dynamics of the COVID-19 pandemic in Europe are limited and most of them focus only on the temperature. Most of them are focused on North or Western Europe. To the best of the authors’ knowledge, the association between meteorological parameters and dynamics of the COVID-19 pandemic in Central-European Countries has not been investigated.

This study aimed to analyze the correlation between meteorological conditions (e.g., temperature, relative humidity, sunshine duration, wind speed) and dynamics of the COVID-19 pandemic in Poland (daily number of laboratory-confirmed COVID-19 cases and the number of COVID-19-related deaths).

## 2. Materials and Methods

### 2.1. Study Area

Poland’s territory covers 312,696 km^2^, of which 98.5% is dry land and 1.5% is water. As of 30 June 2020, the population of Poland amounted to 38,354,000 [25]. The country is relatively flat, located mostly in lowlands and reaches from Carpathian Mountains in the south to the Baltic Sea in the north. The country is in the temperate climatic zone and the territory is influenced mainly by Oceanic (Atlantic) and continental air masses from western and eastern directions, respectively, as well as polar and tropical air from the north and the south, respectively. The highest mean annual temperature is observed in the west and southwest parts of Poland and the lowest in the northeast area and mountains. The northwest of the country as well as the upland and mountainous parts experience the largest annual sum of precipitation and the maximum monthly sum is observed in July and the minimum in February.

The country’s location determines distinct seasonal variations of thermal variables used in the study but other meteorological predictors also reveal a clear annual cycle in Poland. The largest seasonal differences are found for moisture over Eastern Europe [26], and wind speed has an annual cycle with the highest values in winter and the lowest in summer [27]. Bartoszek et al. [28] described monthly sunshine duration climatology for Poland following. On the other hand, daily variability of meteorological variables is strongly influenced by atmospheric circulation and synoptic patterns [29].

The greatest population concentration is in the south and central part of Poland.

### 2.2. Data Collection

**Figure 1:**
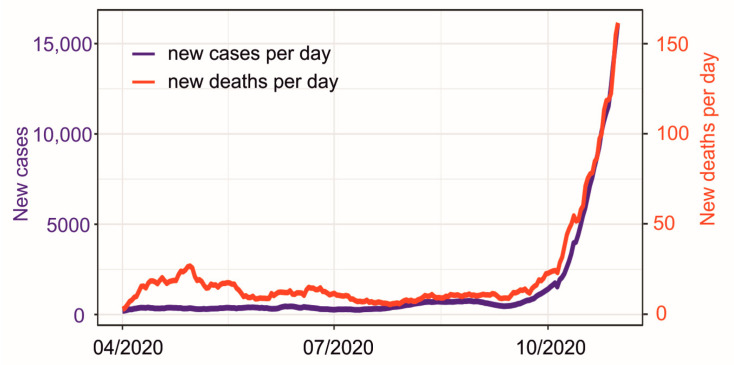
Daily new confirmed Coronavirus disease 2019 (COVID-19) cases (the blue color) and new deaths (the red color). The 7-day moving average is shown.

Data on daily number of laboratory-confirmed COVID-19 cases and the number of COVID-19-related deaths were gatheredfrom the official governmental website of the Polish Ministry of Health [11]. In Poland, diagnosis of COVID-19 is based on the detection of unique sequences of SARS-COV-2 virus ribonucleic acid by real-time reverse-transcription polymerase chain reaction (RT-PCR) [11,12]. This study includes data from April to the end of October 2020. Figure 1 presents smoothed new cases and new deaths for the analyzed period. This time limit was chosen due to the following reasons: (1) since April, wide access to laboratory diagnostics and RT-PCR tests on patients suspected of having COVID-19 has been provided throughout Poland; (2) on 26 October, significant changes in quarantine rules have been implemented; and (3) on 31 October, surveillance definition for COVID-19 was significantly revised and the possibility of using rapid antigen tests has been implemented. The changes in quarantine rules, the COVID-19 case definition and the possibility of performing an antigen test instead of the RT-PCR test could significantly affect the number of laboratory-confirmed COVID-19 cases. For this reason, this study includes data from April to the end of October 2020. Moreover, the period chosen for the analysis took place in order to better detect the effects of the meteorological conditions on the dynamics of COVID-19 pandemic, since inserting data from October onwards into the analysis would make the investigation of the meteorological conditions/COVID-19 relationship unfeasible.

**Figure 2:**
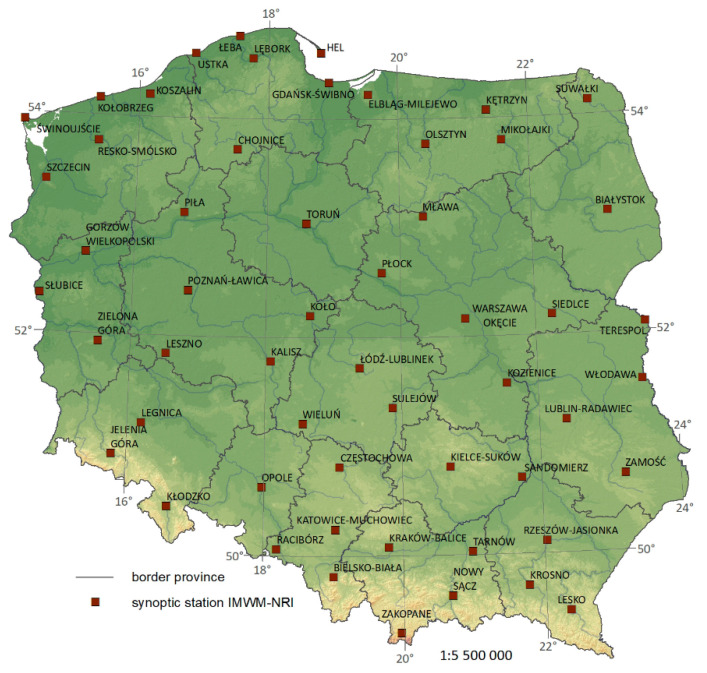
Localization of 55 synoptic stations used in the study.

**Figure 3:**
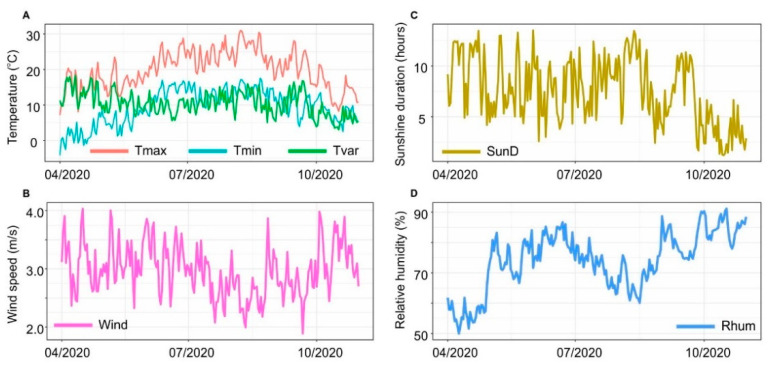
Daily values of maximum temperature (Tmax) (**A**—in red), minimum temperature (Tmin) (**A**—in blue), variability of mean daily temperature (Tvar) (**A**—in green), sunshine duration (SunD) (**C**), wind speed (Wind) (**B**), relative humidity (Rhum) (**D**) for Poland.

In order to assess the influence of meteorological conditions on pandemic dynamics, the meteorological observations from 55 synoptic stations in Poland were used (Figure 2). On their basis we created a dataset which include averaged daily values of maximum temperature, minimum temperature, variability of mean daily temperature, sunshine duration, wind speed, and relative humidity for the whole of Poland. In Figure 3, time series of these parameters are shown.

**Figure 4:**
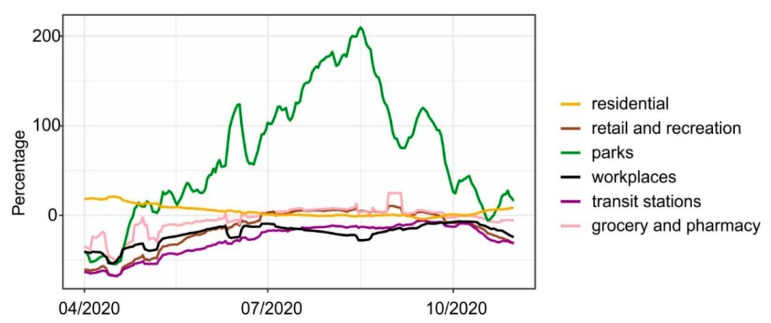
COVID-19 Community Mobile. The raw data were smoothed to the 7-day moving average. The figure presents how the mobility of people has changed compared to the reference period (1 March 2020–2 June 2020).

To perform detailed analyses and discussion of obtained results, we also used the COVID-19 Community Mobility Reports provided by Google [30]. These reports show how movement of people has changed relative to the period before COVID-19 across different categories of places: retail and recreation, grocery and pharmacy, parks, transit stations, workplaces, and residential (Figure 4).

### 2.3. Statistical Analysis

Firstly, the cross-correlation function (CCF) was applied to investigate if any of the meteorological parameters are correlated with new COVID-19 cases or new deaths. CCF is a function of similarity of two time series which give information about the degree of similarity and displacement of one relative to the other [31]. It takes values in the range from −1 (negatively correlated) to 1 (positively correlated). Two series are correlated if the correlation value exceeds the confidence level [32]. In this study, the length of time the data covered is 214 days (from 1 April to 31 October 2020). The variance of the cross-correlation coefficient under the null hypothesis of zero correlation is approximately 1/n for lag k equal 0 where n is the length of the series. The cross-correlation between the two variables is statistically significant at approximately the 5% level of significance—±2/sqrt(n). We assumed that a correlation was significant if it exceededa value of 0.14. It should be note that CCF only gives information about similarity and lag of time. It does not inform whether one variable affects the other or how. For this, additional analyses are needed.

We can assume that a series of new cases and new deaths are dependent on many variables—e.g., meteorological ones. Such an assumption means that all information included in these series can be described by them. However, with many variables we are not able to easily determine which ones carry information and how significant it is in relation to the whole signal. The solution is to use one of the common techniques of multivariate analysis-principal component analysis (PCA). This approach is also used in different areas of human health—e.g., clinical studies [33], schizophrenia [34], health system readiness [35], mortality [36], oral and oropharyngeal cancer [37]. This method was mainly developed for data reduction but also can be efficiently used for finding patterns in the data and to highlight their similarities and differences. PCA can give information about relationship between variables and their contribution to the total variance of the series. One of the main elements of PCA is to determine principal components (PCs) of the data. They can be described as new variables which are mixtures or linear combinations of the initial ones. They capture the correlations between different variables. Moreover, one of the PCA products isalso normalized eigenvectors, whoseelements refer to the response of an individual variable to the considered principal component. A mathematical background of PCA isprovided by Birks [38].

Another technique used in this study was random forest, which is an ensemble machine learning method based on constructing many decision trees. This method combinesa large number of small decision trees into new predictors and therefore is able to make abetter prediction. It also performs better in terms of overfitting to training dataset compared to asingle decision tree method [39]. It is widely used for tasks related to regression and classification in many areas, such as meteorology [40], climatology [41], finance [42], investments, biotechnology [43] and others. By using this method, it is possible to assess which variables have the highest importance in the machine learning model built for representation changes in new COVID-19 cases and deaths. The Meteorologica Al database described above was used as an input to those models that were trained on randomly sampled 70% of data. The remaining 30% was used to validate models. Only default values of tunable parameters from the random Forest R package [44] were tested in this study.

## 3. Results

In this section, the results obtained from all described statistical methods are presented. Each of them was written in a separate subsection. Along with a description of the results obtained, we also presented their interpretation.

### 3.1. Cross-Correlation Function

**Figure 5:**
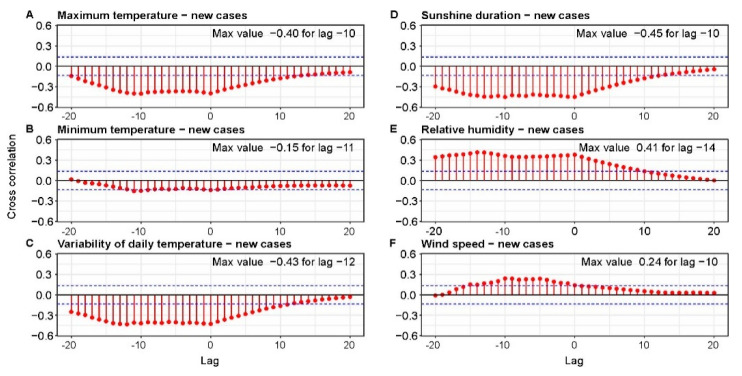
Cross-correlation between number of new cases and meteorology parameters: maximum daily temperature (**A**), minimum daily temperature (**B**), variability of daily temperature (**C**), sunshine duration (**D**), relative humidity (**E**), wind speed (**F**). The 95% confidence bounds are plotted in blue.

CCF was used to find correlation between daily meteorology parameters and epidemiological statistics and to find the time lag of when these signals were most similar. We used averaged daily data from synoptic stations, mobility data and confirmed COVID-19 cases and deaths. In Figure 5, CCFs between meteorological parameters and new cases are presented. The results show that the maximum value of correlation was obtained with a sunshine duration equal to −0.45 with time lag of −10 days. This negative correlation means that decreasingthe number of hours of sunshine increasesthe number of new cases with a 10-day delay. High value of CCF was also obtained for the daily temperature range (−0.43) with a similar time lag. However, it is worth noting that this parameter is highly correlated with sunshine duration (0.82). Based on Figure 5A, it can beseen that also maximum daily temperature had an influence on confirmed cases. A negative correlation equal to −0.40 means that the drop in maximum temperature may have favored the spread of the pandemic. This meteorological parameter is also correlated with sunshine duration (0.46) but less than the temperature range. Figure 5E shows CCF results for relative humidity. A high positive correlation (0.41) and time lag equal to –14 days indicate that an increase in humidity causes an increase in the number of infections. It should be noted that relative humidity is highly negatively correlated with sunshine duration (−0.63) and temperature range (−0.71). However, it is seen that humidity is correlated with new cases with slightly different time lags. In case of minimal temperature and wind speed, the obtained values of CCF are very low and near the confidence limit. Moreover, time lag is not clearly visible. Therefore, based on CCF results, we can state that these two parameters had only a minor influence on new COVID-19 cases.

**Figure 6:**
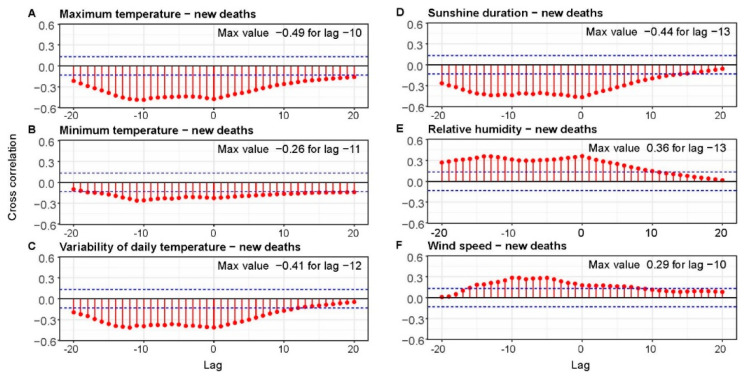
Cross-correlation between number of new deaths and meteorology parameters: maximum daily temperature (**A**), minimum daily temperature (**B**), variability of daily temperature (**C**),sunshine duration (**D**), relative humidity (**E**), wind speed (**F**). The 95% confidence bounds are plotted in blue.

Results of CCF between meteorological parameters and new deaths caused by COVID-19 are presented in Figure 6. In this case, the highest influence on number of deaths was the decreased maximum temperature (negative correlation about −0.49), which occurred 10 days earlier. Additionally, sunshine duration was characterized bya high negative correlation equal to −0.44. However, for this parameter the time lag was −13 days. A similar time lag (−12 days) was achieved for the daily temperature range. For this parameter, the correlation was about −0.41. However, as we mentioned before, it is highly correlatedwith sunshine duration as well as relative humidity, which in this case has a CCF value of 0.36 and a time lag equal −13 days. In case of minimum temperature and wind speed, we have the same conclusions as in the case of correlation with the new cases.

The results of the cross-correlation between meteorological conditions and mobility data are presented in Figures S1–S6 (Supplementary Materials). Higher temperatures favor spending time outdoors in parks or going shopping. It is worth noting, that sunshine duration is almost not correlated with any mobility data. Moreover, the cross-correlations between new COVID-19 cases/deaths and mobility data (Figures S7 and S8) show that mostly there are no significant correlations between them. The exception is mobility data from parks, whose correlations with new cases/deaths were greater than the confidence level and are −0.23 and −0.34, respectively. However, there is no clear peak which makes it difficult to identify lag. In the case of new deaths, a significant correlation was also found for retail and recreation mobility data, but its value was also small (−0.22).

### 3.2. Principal Component Analysis

PCA was computed for sevenvariables: maximum temperature, minimum temperature, sunshine duration, relative humidity, wind speed and new deaths.

Eigenvector elements are dimensionless numbers; therefore, to allow the analysis of the results in a more intuitive way, each eigenvector was divided by its maximum (absolute value) element. Having above in mind, one eigenvector element giving maximal response to the associated Principal Component (PC) is always 100%. This operation results in obtaining “normalized eigenvectors”, whoseelements may reach values between −100 and +100%. Avalue of −100% means the strongest negative correlation of each variable to the estimated PC (variable response to associated PC is high but negative), 100% means direct proportionality (variable response to PC is high and positive) and 0% means absence of statistical dependence (variable not responding to PC). Each individual eigenvector element cannot be interpreted arbitrarily (e.g., we cannot make a statement that 90% means high dependence), but only in relation to the other eigenvalues related to analyzed PC. Each eigenvalue is proportional to the portion of the all input data “variance” that is associated with each eigenvector.

Based on the data presented in Table 1, we can conclude that: (a) the 1st PC represents the vast majority of all data variance and amounts to 85.3% (1 PC is the most statistically significant); (b) new daily deaths considered as a variable is characterized by the greatest positive response to the 1st PC; (c) the second greatest positive response variable to the 1st PC is humidity (14.2%), which means that this variable is the strongest and is statistically dependent to the new daily deaths; (d) the largest negative value of a normalized eigenvector, indicating inverse proportionality is related to variables: the maximum daily temperature and sunshine duration, which areequal to −9.7% and −5.9%, respectively.

By arranging all the series of metrological data listed above together with the series of daily numbers of COVID-19-related deaths in one observation matrix and employing PCA algorithm working on such an input matrix, we obtained results confirming some statistical dependence of the variables. A series of daily number of COVID-19-related deaths respond maximally to the 1st PC, which in turn represents the most significant proportion of the data variance (85.3%). These results allow us to conclude that the most statistically dependent meteorological variable with the number of deaths is relative humidity. Maximum temperature, as well as sunshine duration, ischaracterized by inverse proportionality to the number of COVID-19-related deaths, whichmeans that some effects (occurring over the entire observed time range) seen byincreasing the value of one variable are parallel to decreasing the value of the other variable. Second and 3rd PCs and the next ones are not worth analyzing as the percentage of variance indicates their low statistical significance.

### 3.3. Random Forest

**Figure 7:**
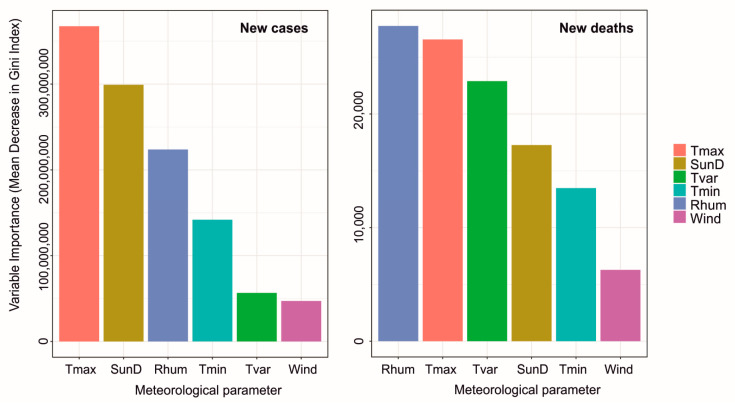
Variables importance from random forest fit. Maximum temperature—Tmax, minimum temperature—Tmin, variability of mean daily temperature—Tvar, sunshine duration—SunD, wind speed—Wind, relative humidity—Rhum.

Results with random forest methods are presented in Table 2. Both new case and new death measuring models built with this machine learning technique predict high correlations (0.95 for new cases and 0.94 for new deaths) and low RMSEs (784 for new cases and 9 for new deaths). For both models, maximum temperature, relative humidity and sunshine duration were the variables which had the greatest impact on prediction. This is seen in Figure 7 where the importance of variables is shown. In case of new deaths, variability of mean daily temperature also had a potential influence.

## 4. Discussion

In this study, we presented results from three methods which gave us information about similarities between series of meteorological parameters and new COVID-19 cases and new deaths (CCF, PCA) or their importance in prediction (random forest). For all methods, we found potential influences on pandemic dynamics were maximum temperature, sunshine duration, relative humidity and variability of mean daily temperature. In the case of new COVID-19 cases, the maximum temperature and sunshine duration had the highest correlation and importance. Increases in temperature and sunshine hours decreased the number of confirmed cases. However, these two parameters are positively correlated each other (0.43), so, based only on these analyses, it is hard to clearly state which factor is dominant: high air temperature or amount of ultraviolent radiation (UV) reaching the Earth’s surface. Relative humidity is also a parameter which had an influence on spread, leading to the pandemic. The occurrence of high humidity caused an increase in the number of COVID-19 cases 14 days later. It is worth notingthat this parameter is weakly correlated with maximum temperature but highly correlated with sunshine duration. This correlation is negative (−0.63), which means that high humidity is when there is a low number of hours of sunshine. Against this background, it is clear that low UV levels and high relative humidity have increase the rate of new COVID-19 cases. We obtained very similar results when analyzing the relationship with new deaths. The statistics look a little different (e.g., correlation values) but the general conclusions are the same. Decreased sunshine duration and increased air humidity had a negative impact on the number of deaths. Our results support the hypothesis of seasonality of SARS-CoV-2 coronavirus infections.

One of the consequences of the pandemic is a drop in mobility, mainly due to the restrictions imposed by national governments. These restrictions are intended to reduce contact between people which should help fight against the pandemic. In this study we also assessedhow meteorological parameters impact on people’s mobility and whether this had an impact on the pandemic dynamic. In Figures S1–S6 (Supplementary Materials) we showed results of CCF between analyzed meteorological parameters and reports about mobility. High correlation is seen between some of them. This applies in particular to the maximum temperature. To determine whetherweather conditions affect mobility and mobility affects COVID-19, or whether weather has a direct impact on the pandemic dynamics, we calculated CCF between new COVID-19 cases/new deaths and mobility. The results presented in Figures S7 and S8 (Supplementary Materials) show that there are no significant correlations between them. Our study showed that, although meteorological parameters were correlated with mobility, their correlation with the dynamics of COVID-19 pandemic was stronger than mobility data. Therefore, we can state that meteorological parameters such as sunshine duration and relative humidity had a direct impact on the dynamic of COVID-19 pandemic in Poland.

We observed that the time lag between meteorological parameters and epidemiological statistics was 10 to 14 days. This is in line with the data on the incubation period of SARS-CoV-2 that vary from 2 to 14 days—on average 5–6 days [2]. Most COVID-19 patients see a doctor when they develop symptoms suggestive of infection with SARS-CoV-2 coronavirus. Moreover, the waiting time for the RT-PCR test result is several days (most of the results are within 48 h). Data on viral biology and COVID-19 diagnosis support the observation of time lag described in this study [3].

In line with previous studies, we observed that increase in temperature and high UV levels mitigates the transmission of COVID-19 [14,15,16]. The UV may affect the COVID-19 pandemic by decreasing the viability of SARS-CoV-2, but also by sunlight-induced vitamin D synthesis in the skin (the major source of vitamin D). There is a scientific debate on the role of Vitamin D in SARS-CoV-2 infections [45]. Some researchers suggest that vitamin D protects against COVID-19 [46].

A study on meteorological factors and the COVID-19 transmission carried out in four cities in China showed that temperature and relative humidity were the main driving factors ofCOVID-19 transmission (with the season and geographic location) [47]. Moreover, the authors observed that the lognormal distribution model had the best fit for the changes of dynamics of the COVID-19 pandemic. Likewise, fitting parameters varied among the four cities. In our study, data for the whole country were analyzed [47]. Astudy from China indicates further research areas—e.g., potential regional differences in the dynamics of COVID-19 pandemic depending on meteorological factors in different administrative parts of Poland.

The data from this study reveal several practical implications. Data from weather forecast models can be used by governments to support the decision-making process to maintain or lift nonpharmaceutical interventions against COVID-19. Moreover, meteorological parameters should be applied in COVID-19 projection and forecasting models. The inclusion of meteorological parameters in COVID-19 projections models can improve the reliability of the obtained data concerning the predicted number of COVID-19 cases and deaths. Moreover, the knowledge of the correlation between the meteorological parameters and the risk of COVID-19 transmission can be used to formulate warning alerts for the local community—e.g., implementation of selected protective behaviors in response to changing meteorological conditions. This is the first study on meteorological parameters and dynamics of the COVID-19 pandemic in Central-European Countries. Further studies are needed to investigate the seasonality of SARS-CoV-2 coronavirus infections, especially in different geographical regions.

This study has several limitations. First, only laboratory-confirmed COVID-19 cases were included in this analysis. We can hypothesize that due to the asymptomatic course of COVID-19, the real number of COVID-19 cases couldbe higher than the laboratory detected figure. Nevertheless, we used data from official governmental announcements that are only available data on the COVID-19 pandemic in Poland. Some authors suggest that all-cause mortality can be used as a reliable estimator of the real COVID-19 death toll [48,49]. It is estimated that deaths attributed to COVID-19 may be underestimated by at least 35% [49]. In Poland, approximately 70,000 excess deaths were reported during the pandemic. It is estimated that 43% of them are caused by COVID-19, and 27% occurred among people previously diagnosed with COVID-19 [11]. This observation is in line with the hypothesis that all-cause mortality should be considered as a reliable estimator of the real COVID-19 death tool. Secondly, synoptic data come from stations usually located outside of most populated areas. On the other hand, cities suffer mostly from pandemic impacts. It is well known that cities experience Urban Heat Island (UHI) effects [50]. These human-induced phenomena modify surface and atmospheric properties in comparison to surrounding nonurban areas. Observed increase inUHI temperature changes atmospheric circulation and influences other wind and humidity patterns. It is also expected that UHI effects will intensify with observed climate change [51].

## 5. Conclusions

We observed a correlation between meteorological parameters and dynamics of the COVID-19 pandemic in Poland. Low temperature, limited sunshine duration as well as high relative humidity increase COVID-19 transmission. Moreover, relative humidity was identified as the most statistically dependent meteorological variable with the number of COVID-19-related deaths. Our study provides important information that may be used by policymakers to support the decision-making process innonpharmaceutical interventions against COVID-19 implemented atlocal and global health levels. Further studies on the meteorological parameters and dynamics of COVID-19 in Central and Eastern Europe are needed to verify the COVID-19 seasonality hypothesis.

## Figures and Tables

**Table 1:** Eigenvalues shown as a percentage of estimated PC variance and normalized eigenvector elements shown as each variable response to the for first three PCs.

Principal Component	1st PC	2nd PC	3rd PC
Eigenvalues [%]	85.3	9.9	3.9
	Normalized eigenvector elements [%]		
Maximum temperature	−9.7	13.3	100.0
Minimum temperature	−4.0	35.4	79.0
Sunshine duration	−5.9	−18.0	21.1
Relative humidity	14.2	100.0	−35.0
Wind speed	0.3	0.1	−4.1
New deaths	100.0	−12.6	19.1

**Table 2:** Details of random forest scores.

Parameter	r	R2	RMSE
new cases—10 days delay	0.95	0.91	784
new deaths—15 days delay	0.94	0.88	9

Abbreviations: r—correlation; R2—square of the correlation coefficient; RMSE—root mean square error.

## Data Availability

Data are available upon reasonable request.

## Supplementary Materials

The following are available online at https://www.mdpi.com/article/10.3390/ijerph18083951/s1, Figure S1. Cross-correlation between maximum temperature and mobility data: (A) retail and recreation, (B) grocery and pharmacy, (C) parks, (D) transit stations, (E) workplaces, (F) residential; Figure S2. Cross-correlation between sunshine duration and mobility data: (A) retail and recreation, (B) grocery and pharmacy, (C) parks, (D) transit stations, (E) workplaces, (F) residential; Figure S3. Cross-correlation between relative humidity and mobility data: (A) retail and recreation, (B) grocery and pharmacy, (C) parks, (D) transit stations, (E) workplaces, (F) residential; Figure S4. Cross-correlation between minimum temperature and mobility data: (A) retail and recreation, (B) grocery and pharmacy, (C) parks, (D) transit stations, (E) workplaces, (F) residential; Figure S5. Cross-correlation between wind speed and mobility data: (A) retail and recreation, (B) grocery and pharmacy, (C) parks, (D) transit stations, (E) workplaces, (F) residential; Figure S6. Cross-correlation between variability of daily temperature and mobility data: (A) retail and recreation, (B) grocery and pharmacy, (C) parks, (D) transit stations, (E) workplaces, (F) residential; Figure S7. Cross-correlation between COVID-19 new cases and mobility data: (A) retail and recreation, (B) grocery and pharmacy, (C) parks, (D) transit stations, (E) workplaces, (F) residential; Figure S8. Cross-correlation between COVID-19 new deaths and mobility data: (A) retail and recreation, (B) grocery and pharmacy, (C) parks, (D) transit stations, (E) workplaces, (F) residential.
