# Supplementary material for: Impact of Meteorological Conditions on the Dynamics of the COVID-19 Pandemic in Poland
Source: Int J Environ Res Public Health. 2021 Apr 9;18(8):3951. doi: 10.3390/ijerph18083951 (PMC8070474; doi:10.3390/ijerph18083951)
Supplement: Supplementary file 1 [file ijerph-18-03951-s001.pdf]

Supplementary material: Impact of meteorological conditions on the dynamics of COVID-19 pandemic in Poland

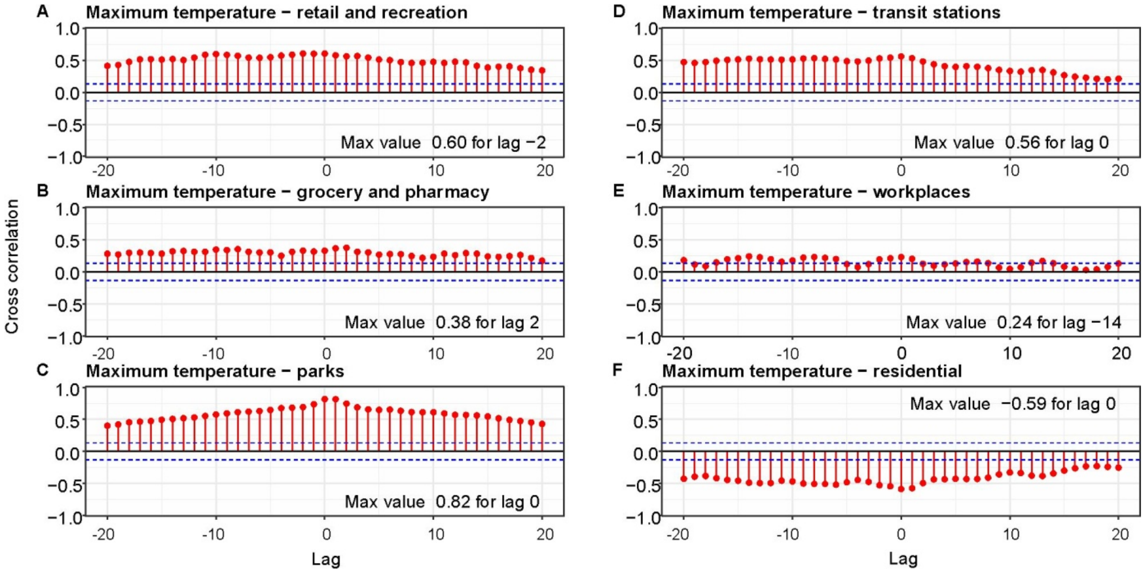

Figure S1. Cross-correlation between maximum temperature and mobility data: (A) retail and recreation, (B) grocery and pharmacy, (C) parks, (D) transit stations, (E) workplaces, (F) residential.

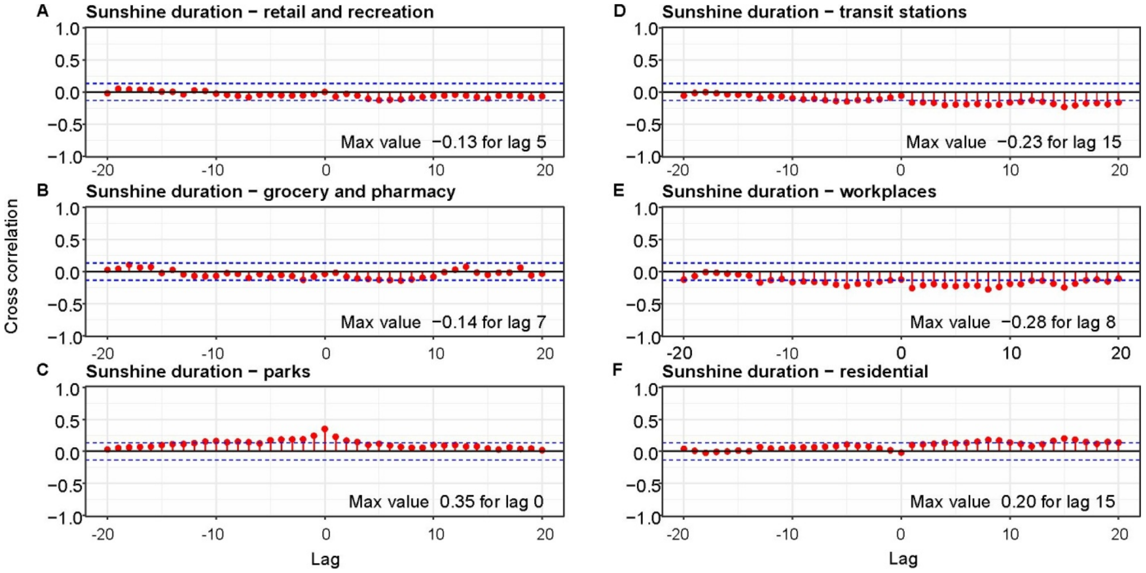

Figure S2. Cross-correlation between sunshine duration and mobility data: (A) retail and recreation, (B) grocery and pharmacy, (C) parks, (D) transit stations, (E) workplaces, (F) residential.

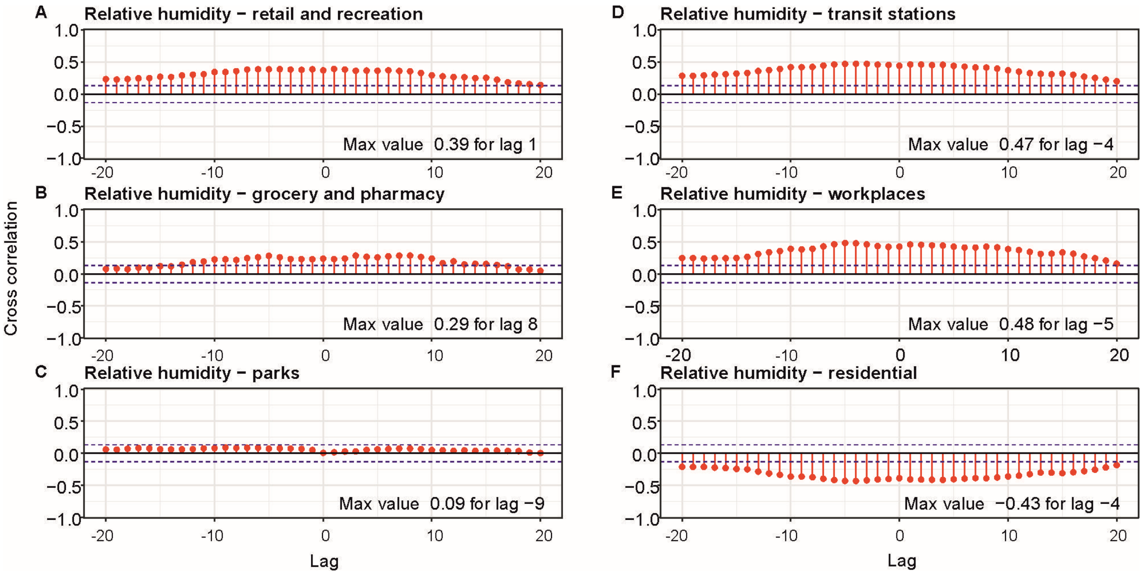

Figure S3. Cross-correlation between relative humidity and mobility data: (A) retail and recreation, (B) grocery and pharmacy, (C) parks, (D) transit stations, (E) workplaces, (F) residential.

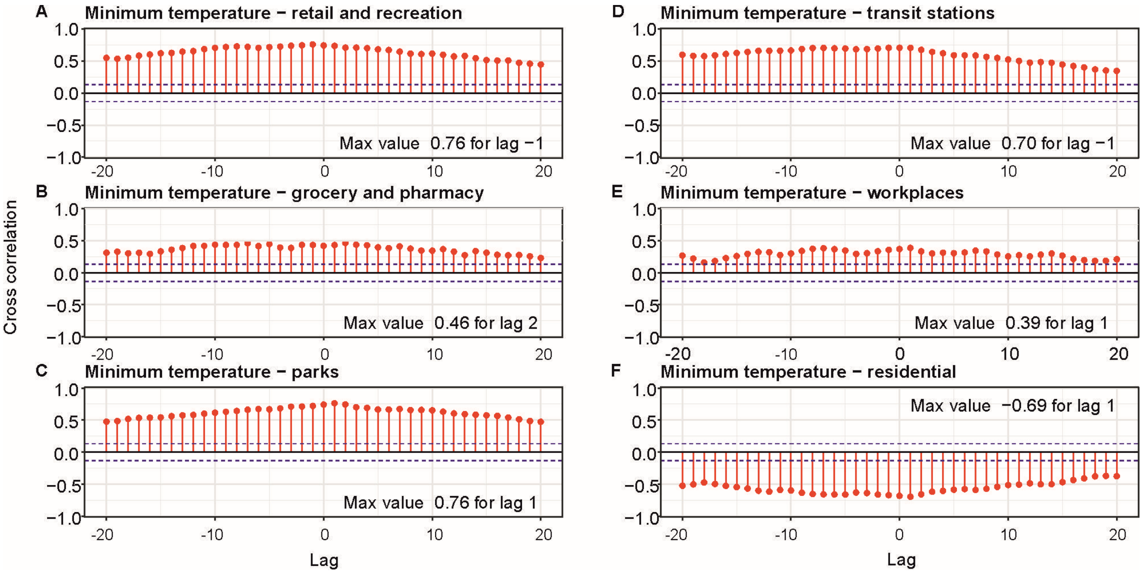

Figure S4. Cross-correlation between minimum temperature and mobility data: (A) retail and recreation, (B) grocery and pharmacy, (C) parks, (D) transit stations, (E) workplaces, (F) residential.

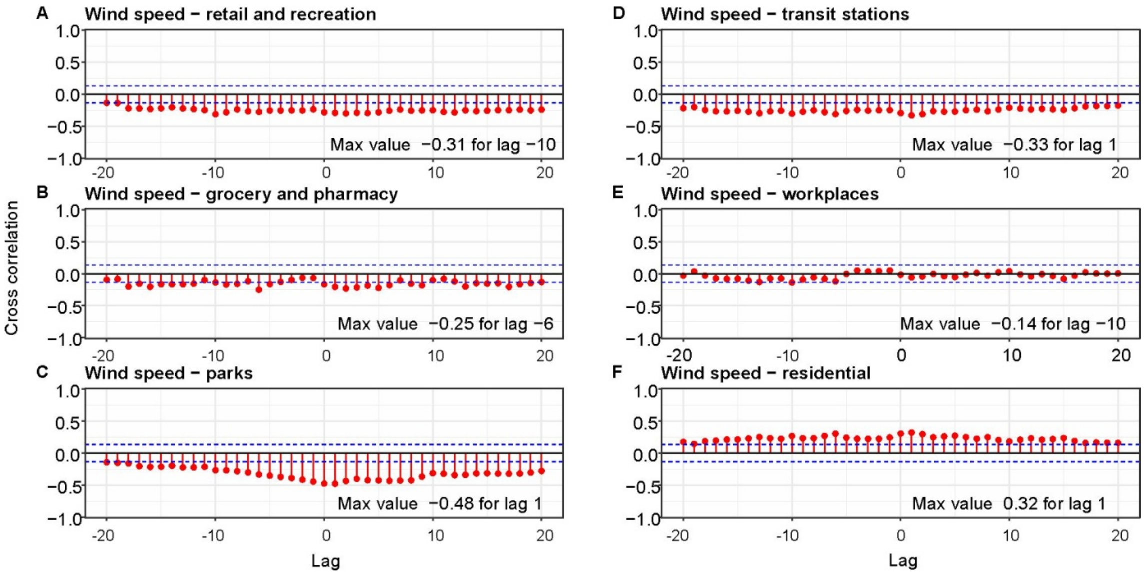

Figure S5. Cross-correlation between wind speed and mobility data: (A) retail and recreation, (B) grocery and pharmacy, (C) parks, (D) transit stations, (E) workplaces, (F) residential.

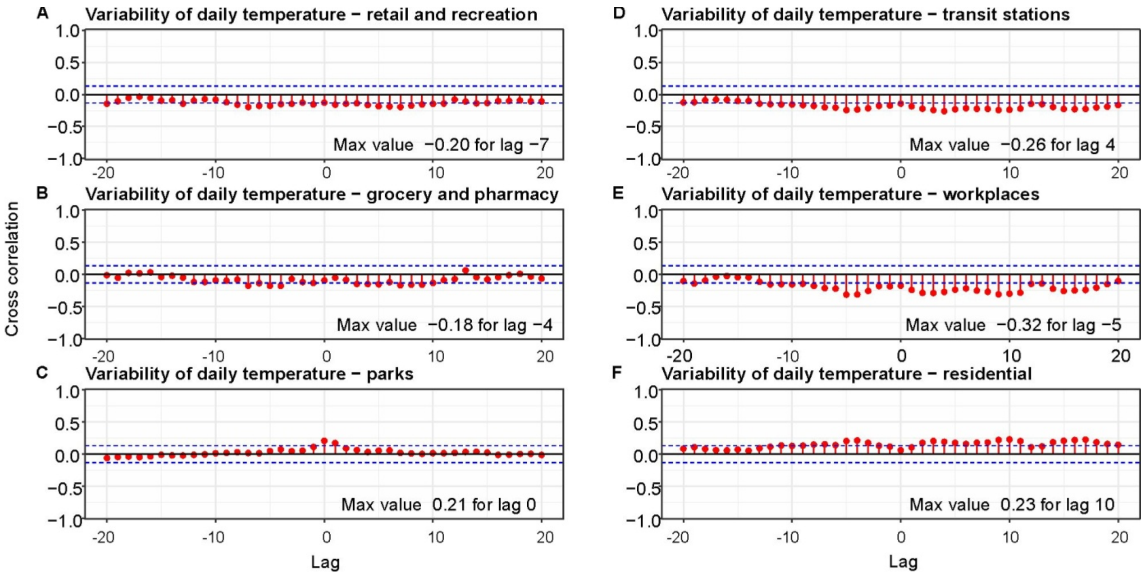

Figure S6. Cross-correlation between variability of daily temperature and mobility data: (A) retail and recreation, (B) grocery and pharmacy, (C) parks, (D) transit stations, (E) workplaces, (F) residential.

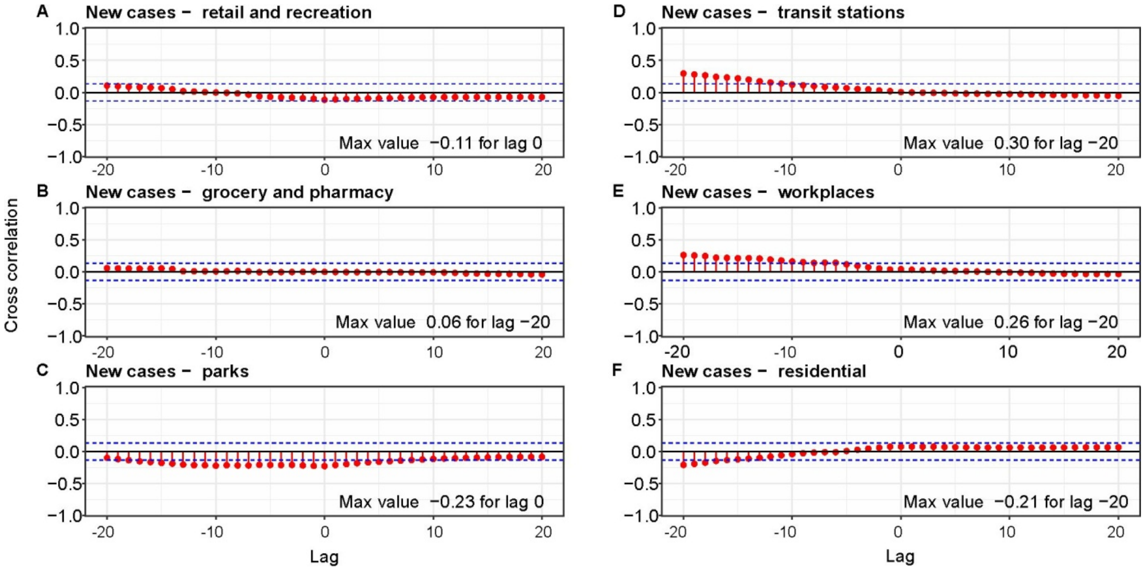

Figure S7. Cross-correlation between COVID-19 new cases and mobility data: (A) retail and recreation, (B) grocery and pharmacy, (C) parks, (D) transit stations, (E) workplaces, (F) residential.

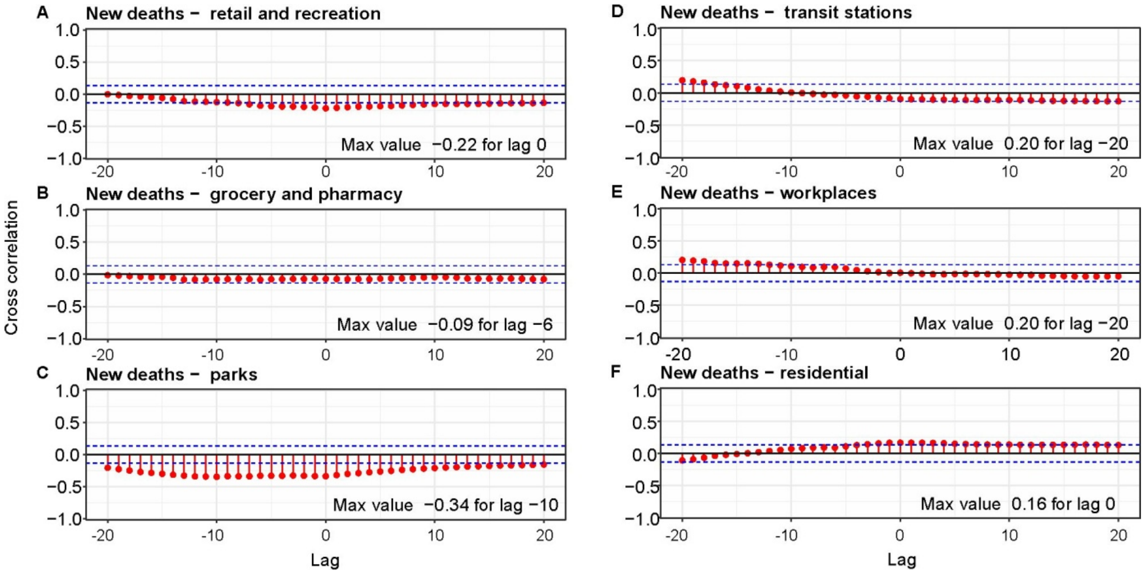

Figure S8. Cross-correlation between COVID-19 new deaths and mobility data: (A) retail and recreation, (B) grocery and pharmacy, (C) parks, (D) transit stations, (E) workplaces, (F) residential.
